# Health Tract, No. 280

**Published:** 1867-03

**Authors:** 


### PASSING AWAY.

Beautiful and bright are the mornings which come to the young, and hours of gladness follow, and thus for successive years, until at length a day comes, the rising of whose sun was as bright as any that ever preceeded it, and yet, before its close, an incident has occurred, almost as unlooked for as a gleam of lightning in a cloudless sky. The unexpected crease has been for the first time noticed in the hitherto polished forehead so faultlessly smooth, and the unwelcome conviction flits across the mind that youth is "passing away." Awhile later, and the cords and veins begin to stand out on the back of the hand, and we instinctively draw it in, as if afraid our friend might also notice that we were "passing away."

Next, the hateful crow-feet disfigure the corners of the eyes; we walk around an obstacle rather than clear it at a bound; we let down the bars rather than scale the fence; we are not so hot for argument as we on e were; we rather sit in silence than contend; we become less uncompromising in our opinions; our assertions are less dogmatic; our invectives less sweeping; we become more considerate; more disposed to "make allowances" for the faults and foibles, and even the crimes of others, as if growing more in unison with the sentiment:

"That mercy I to others show,

That mercy show to me!"

and as if we felt that to the "judgement" we were "passing away.' Then again, a tooth or two has fallen out, and we instinctively take a seat at the window, when about to read the morning paper; we look more for facts, less for opinions; men's characters are measured by their conduct rather than by their professions; we are more anxious to learn what men do, than what they say; and we consider what is in the heart of greater importance than what is in the head. In all our judgements we are more deliberate as we become more sensible that there is less ability and less time to correct mistakes, for that we are "passing away."

The streets are now less full, and so are the churches, of the friends of our school-boy days; of whom in the whirl of business we have regretful thoughts, and feel of some one more distinctly remembered, "O, how I would like to see him again;" or, as to some other one, known to be living, we determine we will write a letter and talk of old times, and make a thousand inquiries about mutual class-mates and friends; but in another hour business engagements crowd in, the letter is never written; and the next we hear—"he is dead." Then comes the feeling, with an overwhelming force, that we also are "passing away!" And so we are, dear reader, but be it our care, that while the physical man is letting go its hold on this mortal life, the spiritual shall grow stronger day by day, rising above the clogs and shackles of the mortal frame preparatory to being disengaged from it altogether; and at the instant of its complete disentanglement, the vision of "the substance of things hoped for" so long, breaks in upon the ravished sight, and we have "passed away'—to Heaven!

